# In Fanconi anemia, impaired accumulation of bone marrow neutrophils during emergency granulopoiesis induces hematopoietic stem cell stress

**DOI:** 10.1016/j.jbc.2024.107548

**Published:** 2024-07-09

**Authors:** Liping Hu, Weiqi Huang, Bin Liu, Elizabeth A. Eklund

**Affiliations:** 1 Division of Hematology-Oncology, Department of Medicine, Northwestern University, Chicago, Illinois, USA; 2 Division of Hematology-Oncology, Department of Medicine, Jesse Brown VA Medical Center, Chicago, Illinois, USA

**Keywords:** cell cycle, DNA repair, innate immunity, inflammasome, neutrophil, DNA damage response, hematopoiesis

## Abstract

Fanconi anemia (FA) is an inherited disorder of DNA repair due to mutation in one of 20+ interrelated genes that repair intrastrand DNA crosslinks and rescue collapsed or stalled replication forks. The most common hematologic abnormality in FA is anemia, but progression to bone marrow failure (BMF), clonal hematopoiesis, or acute myeloid leukemia may also occur. In prior studies, we found that Fanconi DNA repair is required for successful emergency granulopoiesis; the process for rapid neutrophil production during the innate immune response. Specifically, *Fancc*^−/−^ mice did not develop neutrophilia in response to emergency granulopoiesis stimuli, but instead exhibited apoptosis of bone marrow hematopoietic stem cells and differentiating neutrophils. Repeated emergency granulopoiesis challenges induced BMF in most *Fancc*^−/−^ mice, with acute myeloid leukemia in survivors. In contrast, we found equivalent neutrophilia during emergency granulopoiesis in *Fancc*^−/−^*Tp53*^*+/−*^ mice and WT mice, without BMF. Since termination of emergency granulopoiesis is triggered by accumulation of bone marrow neutrophils, we hypothesize neutrophilia protects *Fancc*^*−/−*^*Tp53*^*+/−*^ bone marrow from the stress of a sustained inflammation that is experienced by *Fancc*^−/−^ mice. In the current work, we found that blocking neutrophil accumulation during emergency granulopoiesis led to BMF in *Fancc*^*−/−*^*Tp53*^*+/−*^ mice, consistent with this hypothesis. Blocking neutrophilia during emergency granulopoiesis in *Fancc*^*−/−*^*Tp53*^*+/−*^ mice (but not WT) impaired cell cycle checkpoint activity, also found in *Fancc*^−/−^ mice. Mechanisms for loss of cell cycle checkpoints during infectious disease challenges may define molecular markers of FA progression, or suggest therapeutic targets for bone marrow protection in this disorder.

Fanconi anemia (FA) is an inherited disorder that is due to mutation in one of the 20+ interrelated DNA-repair genes (1, 2). Hematopoietic abnormalities in FA may include bone marrow failure (BMF) in childhood and susceptibility to clonal hematopoiesis or acute myeloid leukemia (AML) with aging (3, 4). Anemia is the first cytopenia to develop, but during an infectious challenge, the neutrophil production is frequently impaired (5, 6). The Fanconi pathway repairs intrastrand DNA-crosslinks and rescues collapsed or stalled replication forks (7, 8, 9). Fanconi D2 and I associate with DNA during S phase, but require activation by the core complex (Fanconi A-C, E-G, and M) to effect repair (5, 10). G2/M arrest was described in FA HSCs after *in vitro* DNA-crosslinker treatment, suggesting intact cell cycle checkpoints under this condition, although other studies indicate the G2/M checkpoint is impaired in FA, or requires Tp53 for activation (11, 12, 13).

Previously, we found acceleration of BMF and clonal progression in *Fancc*^−/−^ mice during repeated episodes of emergency granulopoiesis; the episodic process for rapid neutrophil production during the innate immune response. This process is in contrast to steady state granulopoiesis, which replaces cells lost to normal programmed cell death (14, 15, 16, 17, 18, 19). Emergency and steady state granulopoiesis are regulated by different cytokines and signaling pathways. For example, interleukin-1 beta (IL1β) is essential for emergency, but not steady state, granulopoiesis (15). During infectious challenges, pathogen activated molecular patterns (pamps) activate the Nlrp3 inflammasome, resulting in IL1β production. This induces a ten-fold increase in granulocyte colony stimulating factor (G-CSF) production during emergency granulopoiesis compared to steady state levels of production (14).

Emergency granulopoiesis has three stages; release of mature bone marrow neutrophils, expansion and accelerated differentiation of HSCs and progenitors, and steady state resumption (15, 16, 17, 18, 19). Murine studies suggest that accumulation of neutrophils in the bone marrow triggers termination of emergency granulopoiesis. As infection resolves, signals that stimulate egress of neutrophils from the bone marrow decrease, resulting in neutrophil accumulation (20). Molecular mechanisms to sense neutrophil abundance and terminate “emergency” production are unknown. In mice, IL1β-dependent G-CSF production is required for early HSC expansion during emergency granulopoiesis, and G-CSF (but not IL1β) is required for neutrophil homeostasis at steady state after induction of neutropenia with Ly6G antibody (20). However, the role of neutrophil abundance in termination of emergency granulopoiesis was not directly demonstrated.

The shortened cell cycle during emergency granulopoiesis facilitates rapid neutrophil production, but decreases the time for rescue of collapsed or stalled replication forks. This risks cell cycle progression with DNA damage, but is self-limited during an efficient emergency granulopoiesis response. We found Fanconi DNA repair activity and expression of core proteins (Fanca/c/f) was increased during emergency granulopoiesis, potentially to address this (17, 21).

Established methods to study emergency granulopoiesis in mice include inflammasome activation by intraperitoneal (IP) injection of an adjuvant/antigen combination (*e.g.*, aluminum chloride/ovalbumin (Alum)), or live or heat killed pathogens (22, 23). Such stimuli release bone marrow neutrophils within 24 h, and maximally expand HSCs and differentiating progenitors by 2 weeks, with steady state resumption by four (16, 17, 18, 19). Live pathogens cause death or chronic infection in WT mice, but we found WT mice tolerated 5+ episodes of Alum or heat killed *Candida albicans* injection every 4 weeks without morbidity or mortality (to mimic repeated infectious challenge in human subjects) (16, 17, 18, 19). After injection of Alum or heat killed Candida, we found that *Fancc*^−/−^ and WT mice had an equivalent release of bone marrow neutrophils, and equivalent serum levels of IL1β or G-CSF (17, 19). However, new neutrophils were not subsequently produced by *Fancc*^−/−^ bone marrow, due to apoptosis of HSCs and differentiating progenitors (17, 18, 19). In addition, 80% of *Fancc*^−/−^ mice developed fatal BMF during 3+ challenges, with AML in survivors (17, 18, 19).

Activation of Atr in response to DNA damage results in Tp53-dependent G1/S or G2/M arrest for replication fork rescue or intrastrand cross-links repair, but apoptosis if repair is unsuccessful (24, 25). Phosphorylation of Chk1 by Atr also activates Tp53-independent G1/S, intra-S or G2/M checkpoints (26, 27). Since human FA kindreds with an impaired Atr pathway have delayed BMF, we tested the contribution of Tp53 to failed emergency granulopoiesis with *Fancc*^−/−^*Tp53*^+/−^ mice (18, 28). We found *Fancc*^−/−^*Tp53*^+/−^ mice had an emergency granulopoiesis response that was similar to WT, with equivalent bone marrow and peripheral blood neutrophilia (18). After multiple challenges, apoptosis of HSCs and differentiating progenitors was minimal in *Fancc*^−/−^*Tp53*^+/−^ bone marrow and BMF did not arise (18).

We hypothesize that bone marrow neutrophil accumulation initiates termination of emergency granulopoiesis and relieves HSC stress (19). Lack of neutrophil accumulation in *Fancc*^−/−^ mice sustains this stress through undefined molecular mechanisms. Identifying such FA specific stress mechanisms is the topic of the current investigations.

## Results

### Neutrophil accumulation during emergency granulopoiesis influences bone marrow failure in mice with impaired Fanconi DNA repair

To investigate the contribution of neutrophil accumulation to bone marrow protection during emergency granulopoiesis, we injected mice with Alum to stimulate this process or saline as a steady state control. Some cohorts were also injected with Ly6G antibody (neutrophil-specific clone 1A8) to prevent accumulation of bone marrow neutrophils above steady state levels, but without inducing absolute neutropenia (*versus* isotype control) (22, 23, 29). Mice with neutrophil accumulation during emergency granulopoiesis (*Fancc*^−/−^*Tp53*^+/−^ or WT) were compared to those with impaired neutrophil accumulation (*Fancc*^−/−^, or Ly6G antibody treated *Fancc*^−/−^*Tp53*^+/−^ or WT). Mice were Alum injected every 4 weeks mimicking several infections per year in humans (30). In control experiments, this Ly6G antibody did not alter circulating or bone marrow monocytes or lymphocytes; either during emergency granulopoiesis or steady state.

**Figure 1:**
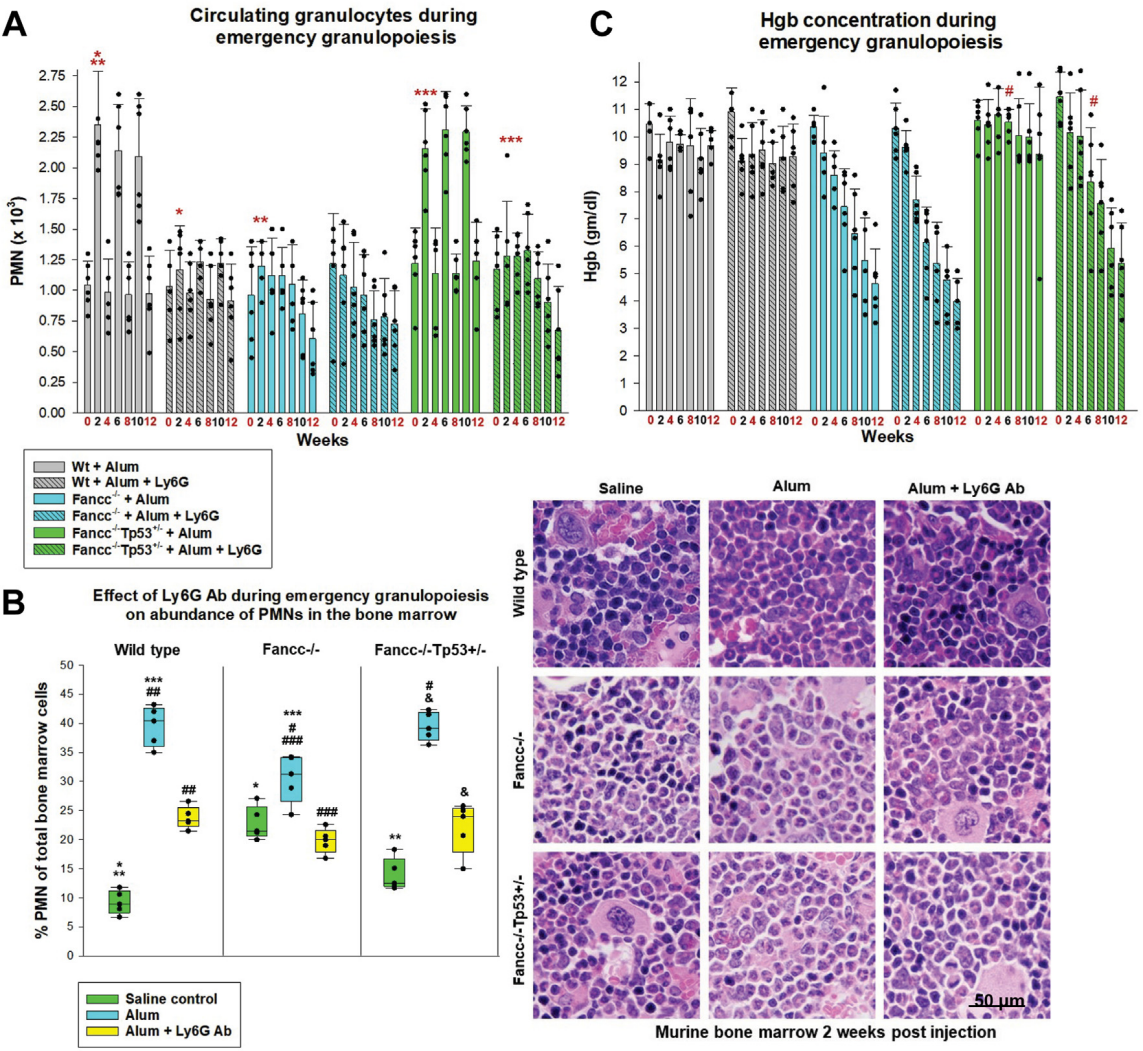
**Blocking bone marrow neutrophil accumulation during emergency granulopoiesis induces cytopenias in Fancc^−/−^Tp53^+/−^ mice.** Fancc^−/−^, Fancc^−/−^Tp53^+/−^ or WT mice were injected IP with Alum to induce emergency granulopoiesis. Some cohorts were injected with Ly6G-antibody to prevent bone marrow neutrophil accumulation (*versus* isotype control antibody). *Red numbers* indicate weeks with injection of Alum or saline (as a steady state control). *A*, Ly6G-antibody treatment induced progressive decrease in peripheral blood neutrophils in Fancc^−/−^Tp53^+/−^ mice during multiple episodes of emergency granulopoiesis. Statistically significant differences in circulating neutrophils indicated by ∗*p* = 0.0005, ∗∗*p* = 0.002 or ∗∗∗*p* = 0.0004. Error bars represent standard deviation and n = 6 for all cohorts. *B*, Ly6G-antibody treatment prevented bone marrow neutrophil accumulation during emergency granulopoiesis, but did not cause absolute neutropenia. Statistically significant differences in bone marrow neutrophils indicated by ∗*p* = 0.0002, ∗∗*p* = 0.014, ∗∗∗*p* = 0.005, #*p* = 0.003, ##*p* = 0.00008, ###*p* = 0.004 or &*p* = 0.0002. Error bars represent standard deviation and n = 6 for all cohorts. Photomicrograph of sternal bone marrow at 40X magnification. *C*, Hgb concentration progressively decreased in Fancc^−/−^Tp53^+/−^ mice treated with Ly6G-antibody during emergency granulopoiesis. Statistically significant differences in Hgb concentration by ^#^*p* = 0.009, n = 6. Error bars represent standard deviation and n = 6 for all cohorts. IP, intraperitoneal.

In control WT mice, we found that treatment with Ly6G antibody maintained baseline levels of circulating or bone marrow neutrophils at 2 weeks after Alum injection (Fig. 1, *A* and *B*). Mild anemia arose during multiple Alum injections in WT mice (compared to saline control, *p* = 0.036 with Ly6G antibody plus Alum, or *p* = 0.001 with Alum alone, n = 6), but there was no significant difference in mice treated with Ly6G antibody *versus* without this treatment in there cohorts (*p* = 0.89, n = 6) (Fig. 1*C*). Platelet counts were not perturbed in either of the WT cohort by Alum injection (not shown).

In *Fancc*^−/−^*Tp53*^+/−^ mice, Ly6G antibody treatment blocked Alum-induced neutrophil increase, as in WT. However, progressive neutropenia arose in *Fancc*^−/−^*Tp53*^+/−^ mice after multiple injections of Alum plus Ly6G antibody (*p* = 0.016, n = 6 at 12 weeks), unlike with Alum alone (Fig. 1, *A* and *B*). Blocking neutrophil accumulation during emergency granulopoiesis also induced a progressive Hgb decline in *Fancc*^−/−^*Tp53*^+/−^ mice (Fig. 1*C*); resulting in relative anemia compared to *Fancc*^−/−^*Tp53*^+/−^ mice injected with Alum alone (*p* = 0.009, n = 6).

Adding Ly6G antibody treatment had little impact on the impaired neutrophil production in *Fancc*^−/−^ mice during emergency granulopoiesis (Fig. 1, *A* and *B*). Progressive neutropenia developed during multiple Alum-induced emergency granulopoiesis episodes in *Fancc*^−/−^ mice and this was not altered by the addition of Ly6G antibody (*p* = 0.002 or *p* = 0.003, respectively, *versus* steady state control, at 12 weeks, n = 6), but was not different between the two cohorts (*p* = 0.057, n = 6 by two-way ANOVA). In *Fancc*^−/−^ mice, Ly6G antibody treatment also did not alter the progressive anemia that occurred over multiple Alum injections (*p* = 0.33, n = 6 by two-way ANOVA) (Fig. 1*C*). Bone marrow neutrophils in these experiments were expressed as a percent of the total bone marrow mononuclear cells. At steady state, this percent was greater in *Fancc*^−/−^ bone marrow compared to WT, consistent with the lower percent of erythroid progenitors in *Fancc*^−/−^ bone marrow, as previously described (19).

We found progressive, absolute neutropenia during emergency granulopoiesis that was similar in *Fancc*^−/−^*Tp53*^+/−^ mice treated with Ly6G antibody and in *Fancc*^−/−^ mice with or without Ly6G (*p* = 0.79, n = 6 by two-way ANOVA). Progressive anemia was also similar in the three cohorts during multiple emergency granulopoiesis episodes (*p* = 0.38, n = 6 by two-way ANOVA).

**Figure 2:**
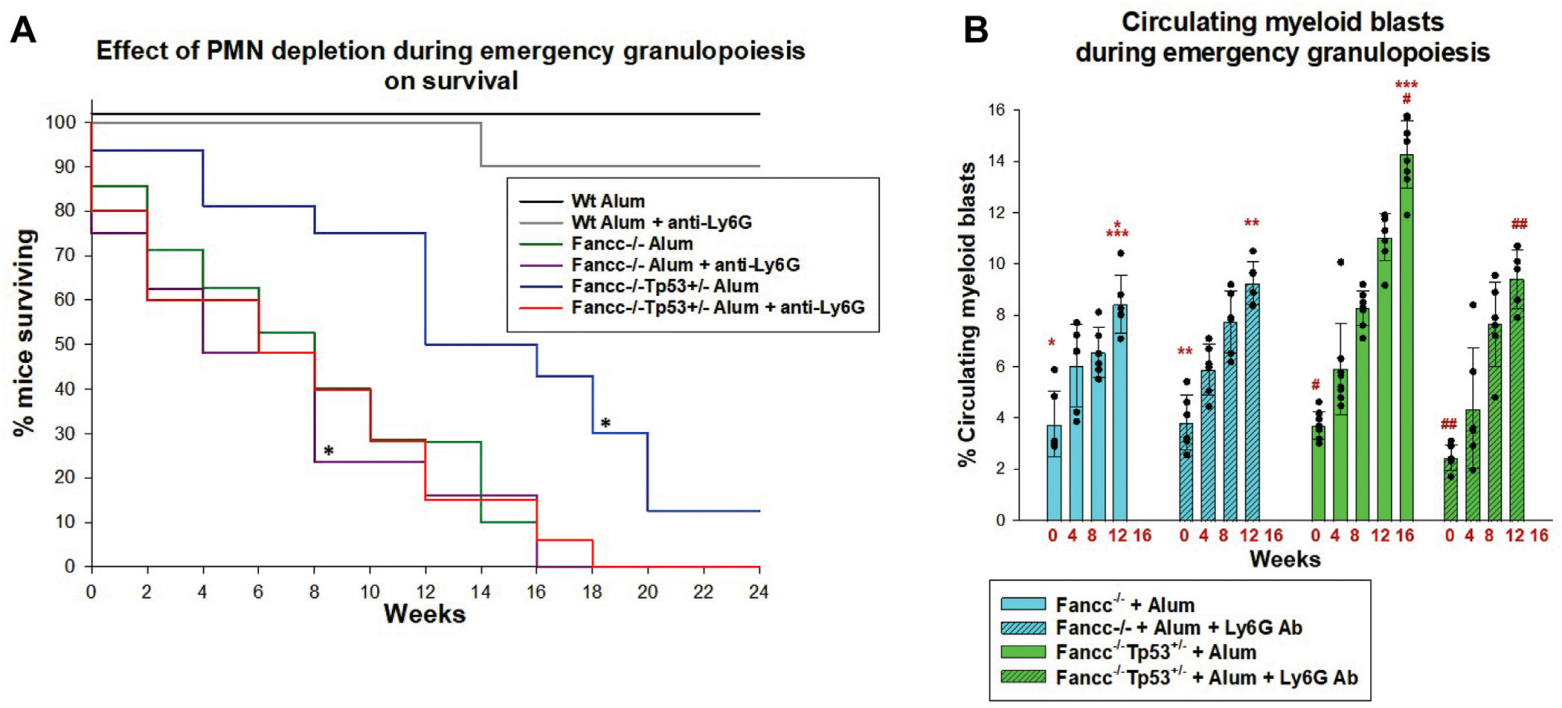
**Blocking bone marrow neutrophil accumulation during emergency granulopoiesis in Fancc**^**−/−**^**Tp53**^**+/−**^**mice shortens survival, but delays leukemogenesis.** Fancc^−/−^, Fancc^−/−^Tp53^+/−^ or WT mice were injected with Alum to induce emergency granulopoiesis, and some cohorts were also injected with Ly6G antibody to prevent neutrophil accumulation during this process. Mice were injected every 4 weeks with Alum injection indicated in *red*. *A*, Ly6G-antibody treatment during emergency granulopoiesis shortened survival of Fancc^−/−^Tp53^+/−^ mice. Statistically significant difference between survival in Fancc^−/−^Tp53^+/−^ mice with Alum *versus* Alum plus Ly6G treatment indicated by ∗*p* = 0.025 with n = 6 for all cohorts. *B*, Ly6G-antibody treatment during emergency granulopoiesis delayed AML in Fancc^−/−^Tp53^+/−^ mice. AML was defined as >10% circulating myeloid blasts. Statistically significant differences indicated by ∗*p* = 0.00006, ∗∗*p* = 0.000032, ∗∗∗*p* = 0.001, ^#^*p* = 0.000026, or ^##^*p* = 0.003. Error bars represent standard deviation and n = 6 for all cohorts. AML, acute myeloid leukemia.

Ly6G-antibody treatment during emergency granulopoiesis shortened survival of *Fancc*^−/−^*Tp53*^+/−^ mice compared to Alum alone (*p* = 0.025, n = 6 by rank sum analysis), but did not alter survival in WT (*p* = 0.56, n = 6 by rank sum analysis) or *Fancc*^−/−^ mice (*p* = 0.64, n = 6 by rank sum) (Fig. 2*A*). Subsequently, 50% of Alum-injected *Fancc*^−/−^*Tp53*^+/−^ mice survived 16 weeks, but this decreased to only 6 weeks in *Fancc*^−/−^*Tp53*^+/−^ mice injected with Alum plus Ly6G antibody. The latter was similar to Alum-injected *Fancc*^−/−^ mice with or without Ly6G treatment (*p* = 0.75, n = 6). All three genotypes survived >16 weeks during control saline injections every 4 weeks (17, 18).

We analyzed these cohorts for BMF *versus* AML (defined as >10% myeloid blasts) as a cause of death. In *Fancc*^−/−^ mice surviving two or more Alum injections, the increase in myeloid blasts was not altered by Ly6G antibody treatment (*p* = 0.19, n = 6 by two-way ANOVA) (Fig. 2*B*). In *Fancc*^−/−^*Tp53*^+/−^ mice, adding Ly6G antibody during multiple Alum injections delayed emergence of circulating myeloid blasts (*p* = 0.003, n = 6 by two-way ANOVA). During episodes of Alum-induced emergency granulopoiesis, the increase in myeloid blasts was equivalent in *Fancc*^−/−^*Tp53*^+/−^ mice treated with Ly6G antibody and *Fancc*^−/−^ mice with or without Ly6G (*p* = 0.26, n = 6 at 12 weeks). We found *Fancc*^−/−^*Tp53*^+/−^ mice died with AML during repeated emergency granulopoiesis episodes, but the addition of Ly6G antibody switched the cause of death to BMF. This is similar to the cause of death in most Alum-injected *Fancc*^−/−^ mice, with or without Ly6G. No WT mice had myeloid blasts during 5+ episodes of Alum-induced emergency granulopoiesis, with or without Ly6G antibody treatment.

**Figure 3:**
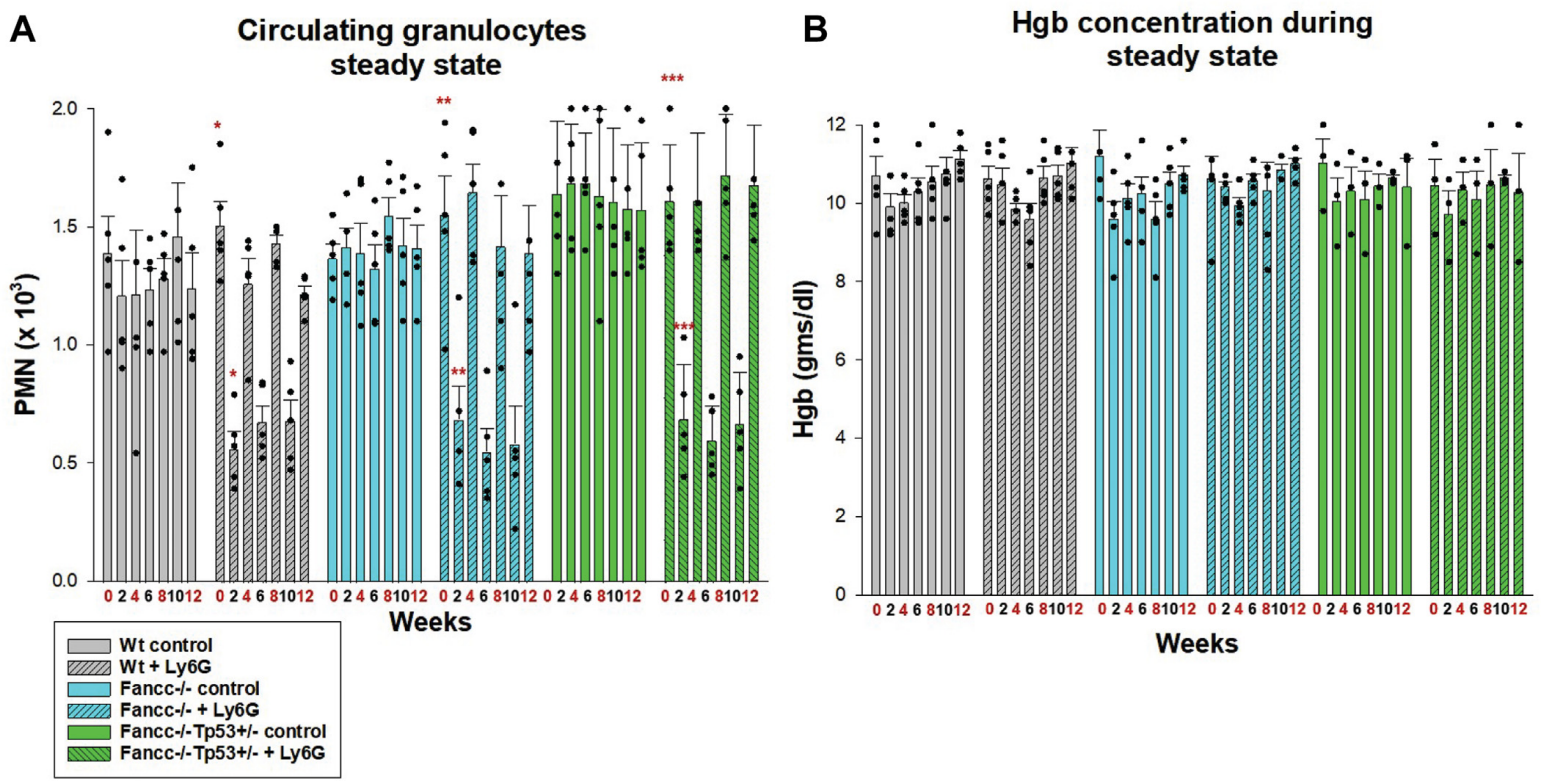
**Episodes of neutropenia at steady state did not induce progressive cytopenias *in Fancc***^***−/−***^**or *Fancc***^***−/−***^***Tp53***^***+/−***^**mice.***Fancc*^−/−^, *Fancc*^−/−^*Tp53*^+/−^ or WT mice were injected with Ly6G antibody every 4 weeks to induce absolute neutropenia at steady state. *Red* numbers indicate weeks with initiation of Ly6G-injection. Statistically significant differences in (*A*) circulating neutrophils or (*B*). Hgb concentration are indicated by ∗*p* = 0.00005, ∗∗*p* = 0.004 or ∗∗∗*p* = 0.0003. Error bars represent standard deviation and n = 6 for all cohorts.

We also investigated the impact of episodes of absolute neutropenia at a steady state on BMF by injecting cohorts of mice with Ly6G antibody alone every 4 weeks. In these studies, we found Ly6G antibody treatment decreased absolute numbers of circulating and bone marrow neutrophils to below steady state in *Fancc*^−/−^, *Fancc*^−/−^*Tp53*^+/−^ or WT mice (Fig. 3*A*). However, neither progressive neutropenia (*p* = 0.62, *p* = 0.43 or *p* = 0.65, respectively, *versus* steady state, n = 6) nor anemia (*p* = 0.53, *p* = 0.89 or *p* = 0.22, respectively, *versus* steady state, n = 6) developed in any cohort over three injection episodes (Fig. 3, *A* and *B*).

### Neutrophil accumulation during emergency granulopoiesis influences apoptosis and DNA damage in bone marrow with impaired Fanconi DNA repair

**Figure 4:**
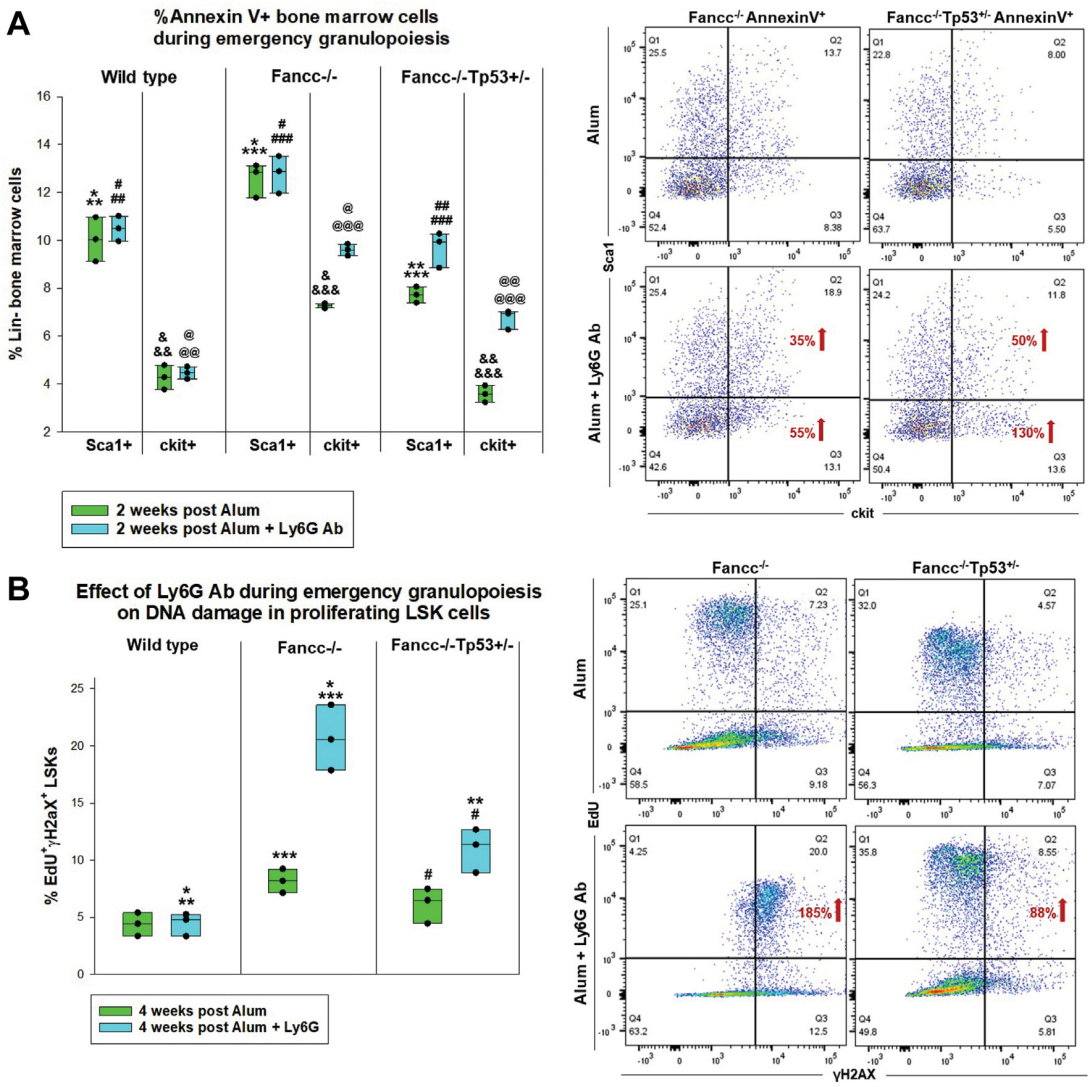
**Blocking bone marrow neutrophil accumulation during emergency granulopoiesis increases bone marrow apoptosis and DNA damage in Fancc^−/−^ or Fancc^−/−^Tp53^+/−^ mice.** Fancc^−/−^, Fancc^−/−^Tp53^+/−^ or WT mice were injected with Alum to induce emergency granulopoiesis. Cohorts were injected with Ly6G antibody to prevent neutrophil accumulation (or isotype control antibody). Mice were injected every 4 weeks and bone marrow was obtained for analysis two or four weeks after the first injection. *A*, Ly6G-antibody treatment during emergency granulopoiesis enhances apoptosis of LSK cells in Fancc^−/−^Tp53^+/−^ mice and Lin-ckit+ cells in Fancc^−/−^ mice. Statistically significant differences indicated by ∗*p* = 0.018, ∗∗*p* = 0.037, ∗∗∗*p* = 0.002, ^#^*p* = 0.0022, ^##^*p* = 0.024, ^###^*p* = 0.008, ^&^*p* = 0.008, ^&&^*p* = 0.033, ^&&&^*p* = 0.007, ^@^*p* = 0.003, ^@@^*p* = 0.005, ^@@@^*p* = 0.008. Error bars represent standard deviation and n = 3 independent experiments for all cohorts. *B*, Ly6G-antibody treatment during emergency granulopoiesis enhances DNA damage in proliferating Fancc^−/−^Tp53^+/−^ or Fancc^−/−^ LSKs. Statistically significant differences indicated by ∗*p* = 0.005, ∗∗*p* = 0.006, ∗∗∗*p* = 0.0002, ^#^*p* = 0.023. Error bars represent standard deviation and n = 3 independent experiments for all cohorts. For both panels, representative histograms are shown with the percent increase with *versus* without Ly6G-antibody indicated. LSK, Lin^-^Sca1^+^ckit^+^.

We next investigated mechanisms for progressive cytopenias during episodes of emergency granulopoiesis in *Fancc*^−/−^ mice and Ly6G antibody treated *Fancc*^−/−^*Tp53*^+/−^ mice. To explore the contribution of apoptosis, we analyzed bone marrow from the cohorts described above by flow cytometry for AnnexinV staining 2 weeks after Alum-injection. This represents the point of maximal neutrophil production in WT mice (17, 18). We found Sca1^+^ cells were overrepresented in the Lin^-^AnnexinV^+^ population from Alum-injected *Fancc*^−/−^ bone marrow compared to WT (*p* = 0.018, n = 3) (Fig. 4*A*). Treatment with Ly6G antibody during emergency granulopoiesis did not alter the abundance of Lin^-^Sca1^+^AnnexinV^+^ bone marrow cells in either genotype (*p* = 0.8 or *p* = 0.6, respectively, n = 3). Lin^-^Sca1^+^AnnexinV^+^ cells were relatively underrepresented in the bone marrow of Alum-injected *Fancc*^−/−^*Tp53*^+/−^ mice compared to Alum-injected *Fancc*^−/−^ mice (*p* = 0.002, n = 3), as anticipated. In *Fancc*^−/−^*Tp53*^+/−^ mice, preventing neutrophil accumulation during emergency granulopoiesis with Ly6G antibody increased apoptosis of Lin^-^Sca1^+^ cells compared to Alum alone (*p* = 0.010, n = 3) (Fig. 4*A*).

Lin^-^Sca1^+^ cells represent an immature progenitor population. To investigate the impact of emergency granulopoiesis episodes on more committed progenitors, we also examined Lin^-^ckit^+^ AnnexinV^+^ cells from these cohorts. Compared to WT, these cells were relatively overrepresented in *Fancc*^−/−^ bone marrow during Alum-induced emergency granulopoiesis (*p* = 0.008, n = 3), but underrepresented in *Fancc*^−/−^*Tp53*^+/−^ bone marrow (*p* = 0.03, n = 3) (Fig. 4*A*). During emergency granulopoiesis, the addition of Ly6G antibody treatment increased Lin^-^ckit^+^AnnexinV^+^ cells in the bone marrow of *Fancc*^−/−^ (*p* = 0.009, n = 3) or *Fancc*^−/−^*Tp53*^+/−^ mice (*p* = 0.005, n = 3), but not WT mice (*p* = 0.88, n = 3). We also found overrepresentation of Lin^-^ckit^+^AnnexinV^+^ cells in the bone marrow of Alum plus Ly6G-treated *Fancc*^−/−^*Tp53*^+/−^ mice *versus* WT (*p* = 0.005, n = 3) (Fig. 4*A*). This suggests impaired Fanconi DNA repair plus impaired accumulation of bone marrow neutrophils during emergency granulopoiesis enhances apoptosis sensitivity, even with Tp53-haploinsufficiency. And has a relatively greater impact on differentiating, compared to more immature, progenitors.

We studied DNA damage in proliferating Lin^-^Sca1^+^ckit^+^ (LSK) cells by flow cytometry for γH2AX and EdU (19, 31). The former marks DNA breaks during S or M phase, and the latter nucleotide incorporation into DNA. We studied bone marrow for persistent damage 4 weeks after Alum-injection, representing steady state resumption in WT mice. Consistent with this, the percent of proliferating WT LSKs at this time point was the same as steady state (*p* = 0.86, n = 3) with minimal DNA damage (Fig. 4*B*). This was not altered by Ly6G antibody treatment of Alum-injected WT mice (*p* = 0.94, n = 3). In *Fancc*^−/−^ mice, the percent of γH2AX^+^EdU^+^ LSKs was greater than WT at 4 weeks after Alum-injection (*p* = 0.0002, n = 3), and this difference was augmented by Ly6G-antibody treatment (*p* = 0.005, n = 3) (Fig. 4*B*). EdU staining intensity of individual *Fancc*^−/−^ LSKs was ∼5 fold less after injection of Alum plus Ly6G *versus* Alum alone; indicating impaired DNA synthesis under the former condition. This was consistent with enhanced DNA damage in *Fancc*^−/−^ mice after Alum plus Ly6G treatment compared to Alum alone.

In *Fancc*^−/−^*Tp53*^+/−^ mice, the percent of γH2AX^+^EdU^+^ LSKs was similar to WT after Alum injection (*p* = 0.07, n = 3), but increased with addition of Ly6G antibody treatment (*p* = 0.006, n = 3 for comparison of the three groups by ANOVA) (Fig. 4*B*). After Alum injection, EdU staining intensity was ∼4 fold less in *Fancc*^−/−^*Tp53*^+/−^ LSKs *versus Fancc*^−/−^ LSKs, suggesting less DNA was synthesized by individual *Fancc*^−/−^ cells with Tp53 haplo-insufficiency during the experiment (Fig. 4*B*). This difference in EdU staining intensity was abrogated by blocking neutrophil accumulation during emergency granulopoiesis in *Fancc*^−/−^*Tp53*^+/−^ mice with Ly6G antibody.

### Neutrophil accumulation during emergency granulopoiesis influences bone marrow transcriptomes

As infection resolves, decreasing signals for neutrophil release result in their accumulation in the bone marrow, triggering termination by unknown mechanisms (20, 22). To investigate molecular mechanisms involved in bone marrow stress during emergency granulopoiesis in FA, we studied the cohorts described above at peak emergency granulopoiesis (2 weeks) or steady state resumption (4 weeks) (17, 18). Bone marrow LSKs from individual mice were isolated for RNA-sequencing, and transcriptomes were subjected to gene specific enrichment analysis with pathway activity suggested by gene ontology (Table 1) (32, 33, 34).

**Table 1:** Gene expression changes during emergency granulopoiesis

Increased 2 weeks	Increased 4 weeks
Cell cycle regulation	Cell cycle regulation
Fbxw9	Fbxw10
Fbxw10	Oga
HSC quiescence	HSC quiescence
Dock9	Dock9
Cdc42bp	Ccng1
Cdc24se	Gfi1
Gfi1	Proliferation
Proliferation	Klf8
Klf8	Cenpv
	Anapc4
Decreased 2 weeks	Innate immune response
Cell cycle regulation	Cebpd
Fbxw11	Csf2ra
Ogt	Tlr4
HSC quiescence	Aldh3b
Rara	Naip2
	Decreased 4 weeks
	Cell cycle regulation
	Fbxw11
	Ogt
	HSC quiescence
	Rara
	Rora
	Gadd45g
	Proliferation
	Klf11
	Innate immune response
	Alox15
	Aldh1a1
	Traf6
	Nfkbia

**Figure 5:**
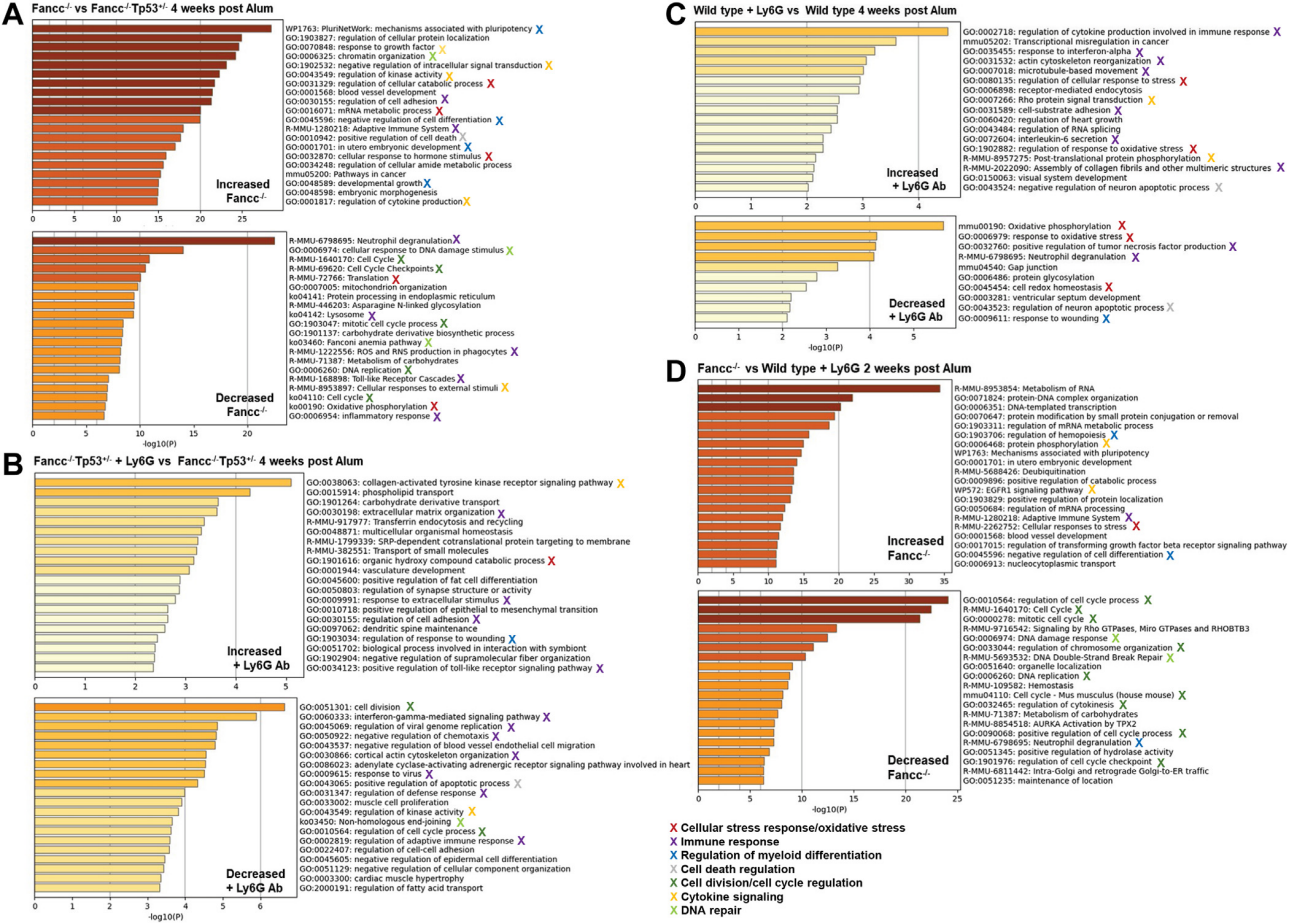
**Transcriptome differences identify common pathway alterations associated with impaired neutrophil accumulation during emergency granulopoiesis.** Fancc^−/−^, Fancc^−/−^Tp53^+/−^ and WT mice were injected with Alum to induce emergency granulopoiesis. Some cohorts were also treated with Ly6G-antibody to block neutrophil accumulation (or isotype antibody control). Mice were sacrificed and bone marrow LSKs isolated two or four weeks after Alum-injection for RNA-sequencing. Transcriptomes were compared and pathway activity suggested by gene ontology. Common pathway differences in various cohort comparisons are indicated by different X colors. *A*, during emergency granulopoiesis, Fancc^−/−^ LSK transcriptomes had decreased activity of pathways involved in phagocyte differentiation and function, the innate immune response, cellular response to stress, oxidative phosphorylation, protein glycosylation and cell cycle regulation in comparison to Fancc^−/−^Tp53^+/−^ LSK transcriptomes, but increased activity of pathways involved in apoptosis, downregulation of cytokine production and growth factor signaling. *B*, during emergency granulopoiesis, Ly6G-antibody treatment of Fancc^−/−^Tp53^+/−^ mice induced LSK transcriptomes with decreased activity of pathways involved in the innate immune response, cellular response to stress, DNA repair and cell cycle regulation, but increased activity of pathways involved in apoptosis *versus* cohorts treated with isotype control antibody. *C*, during emergency granulopoiesis, Ly6G-antibody treatment of WT mice induced LSK transcriptomes with decreased activity of pathways involved in phagocyte differentiation and function, the innate immune response, and cellular response to stress, but increased regulation of cytokine response pathways *versus* cohorts treated with isotype control antibody. *D*, during emergency granulopoiesis, Fancc^−/−^ LSK transcriptomes had increased activity of pathways involved in metabolism of RNA and proteins and Tgfβ signaling, but decreased activity of cell cycle regulatory pathways in comparison to LSK transcriptomes from Ly6G-treated WT mice. LSK, Lin^-^Sca1^+^ckit^+^.

To study the impact of bone marrow neutrophil accumulation during emergency granulopoiesis on FA pathogenesis, we compared *Fancc*^−/−^ and *Fancc*^−/−^*Tp53*^+/−^ mice. Four weeks post Alum injection, we found relatively increased activity of pathways for HSC pluripotency, negative regulation of differentiation, and positive regulation of cell death in *Fancc*^−/−^ LSKs, but decreased expression of neutrophil effector genes (Fig. 5*A*). This suggested apoptosis in *Fancc*^−/−^ bone marrow was associated with ongoing HSC expansion, and that myeloid differentiation was intact in *Fancc*^−/−^*Tp53*^+/−^ mice.

In *Fancc*^−/−^ LSKs, activity of pathways involved in oxidative phosphorylation and N-linked glycosylation were decreased during emergency granulopoiesis compared to *Fancc*^−/−^*Tp53*^+/−^ LSKs. We also found decreased Ogt (O-linked N-acetyl-glucosamine transferase) expression in *Fancc*^−/−^ *versus Fancc*^−/−^*Tp53*^+/−^ LSKs under these conditions. The hexosamine biosynthesis pathway (HBP) produces UDP-N-acetylglucosamine (UDP-GlcNAc) required for both O-linked and N-linked glycosylation (35, 36). A switch from oxidative phosphorylation to aerobic glycolysis may occur during chronic inflammation or other metabolic stresses, antagonizing the HBP (35, 36).

At two and 4 weeks after stimulation of emergency granulopoiesis, cell cycle checkpoint activity was decreased in *Fancc*^−/−^ LSKs compared to *Fancc*^−/−^*Tp53*^+/−^, as indicated by decreased Fbxw11 and increased Fbxw10 expression (E3 ubiquitin ligases) (Table 1) (37, 38). Fbxw10 induces ubiquitination and degradation of Atr, decreasing activation of Chk1 or Tp53 (26, 27, 37). Fbxw11 induces Cdc25 ubiquitination and degradation (38). We also found relatively decreased expression of genes involved in HSC quiescence in LSKs from *Fancc*^−/−^ mice (Table 1), including the cdc42-activator Dock9, and downstream effectors cdc42ce and cdc42bp (39).

To compensate for transcriptome differences due to Tp53 haploinsufficiency rather than neutrophil accumulation during emergency granulopoiesis, we studied LSKs from *Fancc*^−/−^*Tp53*^+/−^ mice with Ly6G antibody treatment *versus* without Ly6G antibody treatment. In *Fancc*^−/−^*Tp53*^+/−^ mice, we found impaired neutrophil accumulation during this process was associated with decreases in HSC quiescence, expression of phagocyte effector genes, and cell cycle checkpoint activity (including Fbxw10 and Fbxw11 alterations) (Fig. 5*B*). As above, we also found decreased Ogt associated with impaired neutrophil accumulation during emergency granulopoiesis.

To identify differences specific to impaired Fanconi DNA repair during the stress of sustained emergency granulopoiesis, we studied the impact of neutrophil accumulation on WT LSK transcriptomes. Similar to the results above, we found Ly6G treatment during emergency granulopoiesis decreased HSC quiescence, phagocyte differentiation, and oxidative phosphorylation pathway activity in WT mice (Fig. 5*C*). We did not find a decrease in cell cycle checkpoint activation or Ogt expression, nor an increase in Fbxw10, but activity of pathways involved in regulating the oxidative stress response was increased.

To clarify stresses specific to impaired Fanconi repair activity at peak emergency granulopoiesis, we compared LSK transcriptomes from Alum-injected *Fancc*^−/−^ mice to those from WT mice injected with Alum plus Ly6G antibody. Dominant differences in *Fancc*^−/−^ LSKs *versus* WT under these conditions included decreased activity of pathways involved in cell cycle regulation, DNA repair, carbohydrate metabolism, and Tgfβ activity in the former, with increased Fbxw10 and decreased Ogt expression (Fig. 5*D*).

**Figure 6:**
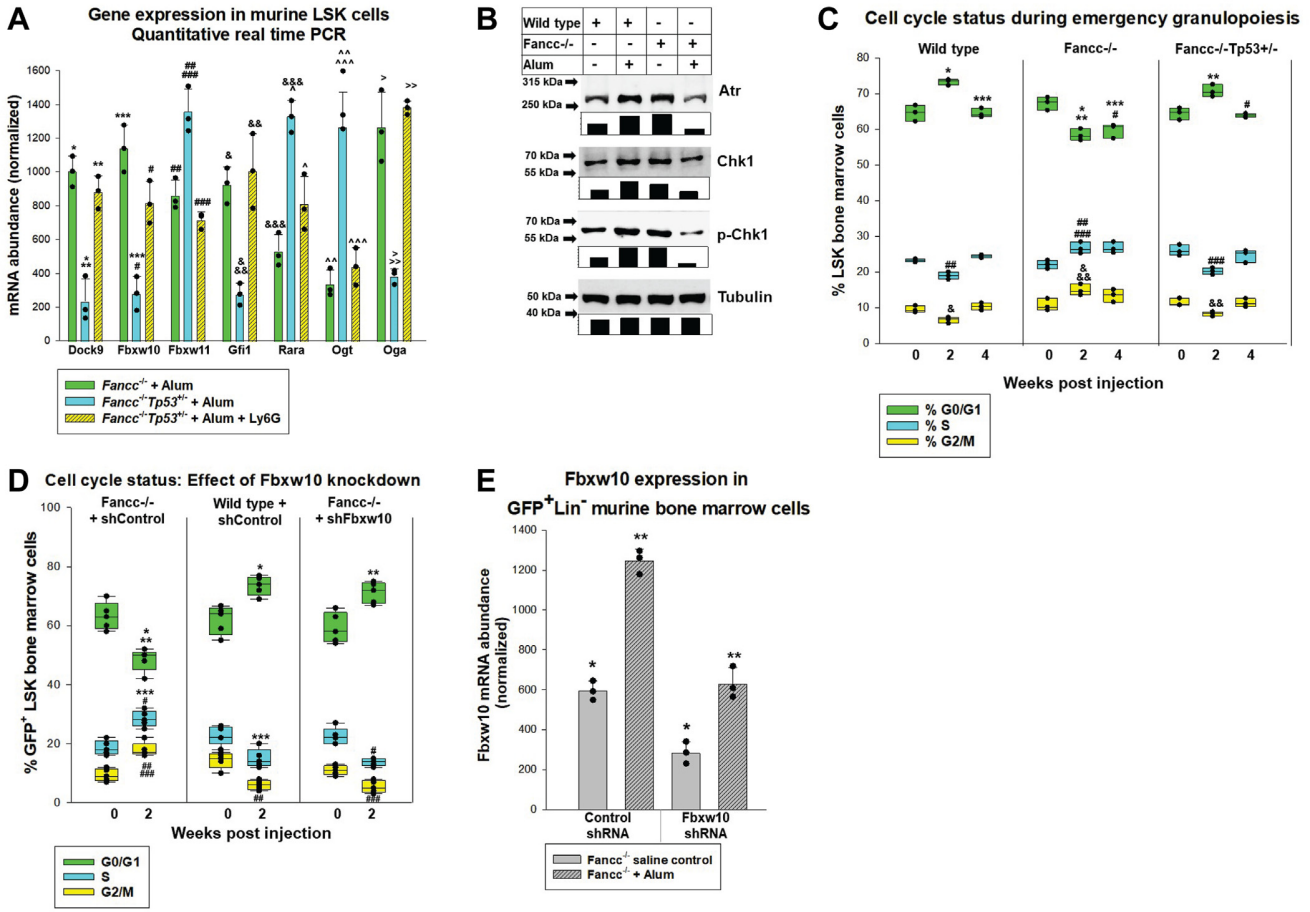
**Cell cycle checkpoints activation in response to DNA damage is impaired during emergency granulopoiesis in Fancc^−/−^ mice.** Fancc^−/−^, Fancc^−/−^Tp53^+/−^ or WT mice were treated with Alum and sacrificed 0, 2, or 4 weeks later for bone marrow analysis. *A*, gene expression differences identified by RNA-sequencing were confirmed in independent experiments. LSKs from Fancc^−/−^, Fancc^−/−^Tp53^+/−^, or Ly6G-treated Fancc^−/−^Tp53^+/−^ mice were isolated 2 weeks after Alum injection and expression of genes identified by RNA-Seq were quantified by real time PCR. Statistically significant differences are indicated by ∗*p* = 0.018, ∗∗*p* = 0.013, ∗∗∗*p* = 0.004, ^#^*p* = 0.001, ^##^*p* = 0.006, ^###^*p* = 0.002, ^&^*p* = 0.0008, ^&&^*p* = 0.005, ^&&&^*p* = 0.0005, ^∧^*p* = 0.009, ^∧∧^*p* = 0.004, ^∧∧∧^*p* = 0.005, ^>^*p* = 0.002, ^>>^*p* = 0.007, or ^>>>^*p* = 0.0004. Error bars represent standard deviation and n = 3 independent experiments for all cohorts. *B*, Atr and phospho-Chk1 are decreased in Fancc^−/−^ bone marrow during emergency granulopoiesis compared to WT. Bone marrow Lin-cells from Fancc^−/−^ or WT mice were obtained 2 weeks after injection of Alum or saline. Western blots of cell lysates were serially probed with antibodies to Atr, phospho-Chk1, total Chk1, or Tubulin (loading control). Protein expression was quantified by densitometry. *C*, during emergency granulopoiesis, Fancc^−/−^ bone marrow LSKs exhibit a relative increase in S phase, but decrease in G0/G1 or G2/M, compared to LSKs from Fancc^−/−^Tp53^+/−^ or WT mice. Cell cycle analysis was performed on LSK cells 0, 2, and 4 weeks post Alum injection and expressed as percent total LSKs. Statistically significant differences indicated by ∗*p* = 0.0001, ∗∗*p* = 0.0008, ∗∗∗*p* = 0.004, ^#^*p* = 0.02, ^##^*p* = 0.002, ^###^*p* = 0.007, ^&^*p* = 0.001, or ^&&^*p* = 0.002. Error bars represent standard deviation and n = 3 independent experiments for all cohorts. *D*, Fbxw10-knockdown in Fancc^−/−^ bone marrow increases cells in G1/G0, and decreases those S and G2/M, during emergency granulopoiesis. Fancc^−/−^ or WT mice were injected with Alum to induce emergency granulopoiesis or saline as a steady state control. Bone marrow harvested 2 weeks later was transduced with vectors to express Fbxw10-specific shRNAs *versus* scrambled control shRNAs, followed by cell cycle analysis of GFP+LSKs. Statistically significant differences indicated by ∗*p* = 0.00003, ∗∗*p* = 0.00001, ∗∗∗*p* = 0.0001, ^#^*p* = 0.00004, ^##^*p* = 0.00012, ^###^*p* = 0.00018. Error bars represent standard deviation and n = 5 independent experiments for all cohorts. *E*, transduction of Fancc^−/−^ bone marrow cells with Fbxw10-specific shRNAs decreases Fbxw10 mRNA during emergency granulopoiesis. GFP+Lin-murine bone marrow cells from the experiment above were analyzed for Fbxw10 mRNA abundance by quantitative real time PCR. Statistically significant differences indicated by ∗*p* = 0.001 or ∗∗*p* = 0.0003. Error bars represent standard deviation and n = 3 independent experiments for all cohorts. LSK, Lin^-^Sca1^+^ckit^+^.

We confirmed key RNA sequencing results in independent experiments with LSKs isolated from *Fancc*^−/−^ or *Fancc*^−/−^*Tp53*^+/−^ mice 2 weeks post Alum-induced emergency granulopoiesis. Some *Fancc*^−/−^*Tp53*^+/−^ mice were also treated with Ly6G antibody. We found increased expression of Fbxw10, but decreased Fbxw11, in Alum-injected *Fancc*^−/−^ or Alum plus Ly6G-antibody treated *Fancc*^−/−^*Tp53*^+/−^ mice compared to Alum-injected *Fancc*^−/−^*Tp53*^+/−^ mice (by real time PCR) (Fig. 6*A*). Expression of these genes was not different in the two cohorts with impaired neutrophil accumulation (*p* = 0.05 or *p* = 0.07 for Fbxw10 or Fbxw11, respectively, n = 3). Similarly, we confirmed increased Dock9 or Gfi1 in LSKs from Alum-injected *Fancc*^−/−^ mice or Alum plus Ly6G-injected *Fancc*^−/−^*Tp53*^+/−^ mice compared to Alum-injected *Fancc*^−/−^*Tp53*^+/−^ mice. Expression of these genes was not different in the cohorts with impaired neutrophil accumulation during this process (*p* = 0.19 or *p* = 0.59 for Dock9 and Gfi1, respectively, n = 3). Expression of retinoic acid receptor alpha was the inverse, consistent with RNA-Seq results (Fig. 6*A*).

We also confirmed RNA sequencing results for Ogt, and investigated expression of the Ogt antagonist, Oga (O-GlcNAcase). Compared to Alum-injected *Fancc*^−/−^*Tp53*^+/−^ mice, we found less Ogt expression in bone marrow LSKs from Alum-injected *Fancc*^−/−^ mice or Alum plus Ly6G-injected *Fancc*^−/−^*Tp53*^+/−^ mice (*p* ≤ 0.001, n = 3 for both comparisons), but more Oga (*p* = 0.002 or *p* < 0.001 respectively, n = 3). We also compared Ogt expression at steady state and post Alum injection in LSKs from these mice. At 4 weeks post Alum injection, we found a modest increase in Ogt expression in WT bone marrow that was not altered by Ly6G-antibody treatment (26.3% ± 6.8% or 23.5% ± 7.3% increase respectively, *p* = 0.100, n = 3 for comparison of the two). Results were similar in *Fancc*^−/−^*Tp53*^+/−^ mice (27.0% ± 5.9% increase, *p* = 0.67 for comparison of the 3 groups by ANOVA, n = 3). Conversely, Ogt mRNA decreased during emergency granulopoiesis in *Fancc*^−/−^ or Ly6G-antibody treated *Fancc*^−/−^*Tp53*^+/−^ mice (56.6% ± 6.2% or 48.1% ± 6.5% decrease respectively, *p* = 0.11 for comparison of the two, n = 3).

We investigated potential downstream mediators of Fbxw10 in Lin^-^ cells from *Fancc*^−/−^ or WT bone marrow 2 weeks after Alum or saline injection. In WT cells, Atr protein increased at peak emergency granulopoiesis, with an accompanying increase in phospho (active) Chk1. Total Chk1 protein increased during emergency granulopoiesis, but the relative abundance of phospho/total Chk1 also increased. Conversely, Atr protein and activated Chk1 decreased relative to steady state in Lin^-^ *Fancc*^−/−^ cells post Alum-injection. Atr and Chk1 mRNA were not altered in either genotype during emergency granulopoiesis (not shown).

### Neutrophil accumulation during emergency granulopoiesis influences cycle checkpoints in mice with impaired Fanconi DNA repair

Based on these results, we investigated cell cycle status of bone marrow LSKs during emergency granulopoiesis (by flow cytometry for DNA content) (Fig. 6*C*). At steady state, we found no difference in the percent of LSKs in G0/G1 (*p* = 0.17 comparing the three by ANOVA, n = 3) or G2/M (*p* = 0.27, n = 3) from WT, *Fancc*^−/−^ or *Fancc*^−/−^*Tp53*^+/−^ mice, with slightly more *Fancc*^−/−^*Tp53*^+/−^ LSKs in S phase (*p* = 0.05, n = 3) (Fig. 6*C*).

Two weeks after Alum injection, the percent of LSKs in G0/G1 increased relative to steady state in WT or *Fancc*^−/−^*Tp53*^+/−^ mice (*p* = 0.032 or *p* = 0.011, respectively, n = 3), but decreased in *Fancc*^−/−^ mice (*p* = 0.003, n = 3) (Fig. 6*C*). There was no difference between the percent LSKs in G0/G1 in *Fancc*^−/−^*Tp53*^+/−^ *versus* WT mice (*p* = 0.68, n = 3). At 4 weeks, this decrease in *Fancc*^−/−^ LSKs in G0/G1 persisted (*p* = 0.012 comparing the three groups by ANOVA, n = 3), but the percent G0/G1 LSKs from *Fancc*^−/−^*Tp53*^+/−^ or WT mice returned to steady state.

Decreased G0/G1 in *Fancc*^−/−^ LSKs may represent an impaired G1/S checkpoint during EG. Consistent with this, the percent of *Fancc*^−/−^ LSKs in S phase increased 2 weeks after Alum injection relative to steady state (*p* = 0.0021, n = 3), but decreased in WT or *Fancc*^−/−^*Tp53*^+/−^ mice (*p* = 0.001 or *p* = 0.002, respectively, n = 3) (Fig. 6*C*). Relatively increased EdU staining intensity of *Fancc*^−/−^ *versus Fancc*^−/−^*Tp53*^+/−^ LSKs during emergency granulopoiesis suggested S phase increase in the former was not due to intra-S arrest (Fig. 4*B*). At 4 weeks, the percent of LSKs in S phase from *Fancc*^−/−^*Tp53*^+/−^ or WT bone marrow returned to baseline (*p* = 0.003 or 0.0017, respectively, two *versus* 4 weeks, n = 3), but S phase increase in *Fancc*^−/−^ LSKs was sustained (*p* = 0.42 two *versus* 4 weeks, n = 3) (Fig. 6*C*).

Two weeks after Alum injection, the percent of *Fancc*^−/−^ LSKs in G2/M rose significantly relative to steady state (*p* = 0.032, n = 3), but decreased in WT or *Fancc*^−/−^*Tp53*^+/−^ LSKs (*p* = 0.004 or *p* = 0.013, respectively, n = 3) (Fig. 6*C*). The percent of LSKs in G2/M returned to steady state levels by 4 weeks in WT, *Fancc*^−/−^*Tp53*^+/−^ and *Fancc*^−/−^ mice (*p* = 0.36, *p* = 0.90, or *p* = 0.062, respectively, for 4 weeks *versus* untreated, n = 3).

We next investigated the contribution of Fbxw10 to the cell cycle abnormalities in *Fancc*^−/−^ bone marrow during emergency granulopoiesis. For these studies, we injected WT or *Fancc*^−/−^ mice with Alum to induce emergency granulopoiesis or saline as a steady state control. Bone marrow was harvested 2 weeks after injection based on the timing of increased Fbxw10 during emergency granulopoiesis in *Fancc*^−/−^ mice. Bone marrow Lin^-^ cells were transduced with a vector to express Fbxw10-specific shRNAs or scrambled control (coexpressing GFP). GFP^+^Lin^-^ckit^+^ cells were analyzed for cell cycle status 24 h post transduction.

At this time point, cell cycle status in Alum-injected *Fancc*^−/−^ or WT cells transduced with scrambled control shRNA was similar to nontransduced cells (compare Fig. 6, *C* and *D*). However, in *Fancc*^−/−^ LSKs transduced with a vector to knockdown Fbxw10, the percent of G0/G1 cells was significantly greater than in *Fancc*^−/−^ LSKs transduced with control vector (*p* = 0.00001, n = 5), and was not significantly different than control vector transduced WT LSKs (*p* = 0.8, n = 5). Fbxw10-knockdown decreased the percent of *Fancc*^−/−^ LSKs in S phase 2 weeks after Alum injection compared to *Fancc*^−/−^ LSKs transduced with vector control (*p* = 0.0001, n = 5), and was not significantly different than control vector-transduced WT LSKs (*p* = 0.09, n = 5). (Fig. 6*D*). Transduction of *Fancc*^−/−^ bone marrow cells with Fbxw10 shRNA vectors also decreased percent LSKs in G2/M compared to control vector (*p* = 0.00018, n = 5), and cells in G2/M were not significantly different than WT control shRNA transduced cells (*p* = 0.6, n = 5). Knockdown of Fbxw10 was >50% in these studies (Fig. 6*E*).

## Discussion

Mice with engineered disruption of Fanconi DNA repair genes do not develop BMF or AML at steady state, but the protected environment of the animal facility does not duplicate ongoing infectious challenges experienced by human FA patients. To consider the impact of such challenges on hematopoiesis, we studied *Fancc*^−/−^ mice during experimentally-induced episodes of emergency granulopoiesis at a frequency mimicking several human infections per year (17, 19). We found emergency granulopoiesis stimuli failed to induce neutrophilia in *Fancc*^−/−^ mice, but most of these mice developed apoptosis of bone marrow HSCs and differentiating progenitors with fatal BMF after several episodes (17, 18, 19). In contrast, episodes of absolute neutropenia in *Fancc*^−/−^ mice at steady state, induced by Ly6G antibody (neutrophil specific clone 1A8), did not result in BMF. This suggests Fanconi DNA repair preferentially protects the genome during the genotoxic stress of the innate immune response rather than playing a role in steady state neutrophil homeostasis.

In response to ssDNA damage, Atr activates Tp53 for p21-induced G1/S or G2/M checkpoint activation, with apoptosis if repair is unsuccessful (24, 25, 26). Atr also activates Chk1 for Tp53-independent activation of G1/S, intra-S and G2/M checkpoints (27). And, activated Atr cooperates with Fanconi DNA repair proteins to rescue replication forks (40). Prior studies suggested that Tp53 is required for G2/M checkpoint activation in FA, potentially preventing HSC exhaustion (13). Results of studies investigating a role for the Fanconi pathway in G2/M arrest were variable, perhaps due to differences in cell types, *in vitro* culture systems, or physiologic conditions (11, 41). Other studies identified a role for the Fanconi DNA repair activity in late S phase arrest (12).

We previously found that Atr and Tp53 were activated in *Fancc*^−/−^ bone marrow cells during emergency granulopoiesis and therefore we studied these mice for the impact of Tp53 on BMF in these mice (17, 18). In response to emergency granulopoiesis stimuli, we found equivalent neutrophil accumulation in the bone marrow and circulation of *Fancc*^−/−^*Tp53*^+/−^ and WT mice, without progressive BMF (18). The current studies investigate related mechanisms.

Accumulation of bone marrow neutrophils is hypothesized to trigger termination of emergency granulopoiesis, potentially protecting *Fancc*^−/−^*Tp53*^+/−^ bone marrow from the stress of sustained attempts to expand HSCs and progenitors (20). Consistent with a role for neutrophil accumulation in bone marrow protection, we found treatment of *Fancc*^−/−^*Tp53*^+/−^ with Ly6G (clone 1A8) antibody during emergency granulopoiesis-induced BMF and survival that were similar to Alum-injected *Fancc*^−/−^ mice. Under our experimental conditions, Ly6G-antibody blocked bone marrow neutrophil accumulation without inducing absolute neutropenia. Although Ly6G clone 1A8 antibody is neutrophil specific, and did not influence abundance of monocytes or lymphocytes in our experiments, off target effects are a possible consideration for additional study.

A sustained innate immune response, due to impaired neutrophil accumulation during emergency granulopoiesis, might sustain production of inflammatory cytokines, with consequent specific damage to FA bone marrow. However, we did not find an increase in the production of inflammatory cytokines or activation of associated signaling pathways in comparisons of *Fancc*^−/−^ to *Fancc*^−/−^*Tp53*^+/−^ LSKs, or LSKs from *Fancc*^−/−^*Tp53*^+/−^ mice with Ly6G antibody treatment *versus* without Ly6G antibody treatment during emergency granulopoiesis. Alternatively, Tp53-haploinsufficiency in *Fancc*^−/−^ bone marrow might decrease inflammatory cytokine production. We found that adding Ly6G-treatment during emergency granulopoiesis decreased activity of interferon gamma pathways in LSK transcriptomes from *Fancc*^−/−^*Tp53*^+/−^ mice compared to Alum alone. Since similar alteration of interferon gamma signaling was not found in comparison of *Fancc*^−/−^ *versus Fancc*^−/−^*Tp53*^+/−^ LSK transcriptomes, this may be specific to differentiating *Fancc*^−/−^*Tp53*^+/−^ LSKs. *Fancc*^−/−^*Tp53*^+/−^ neutrophils were morphologically unremarkable, but impaired neutrophil function may be found in acquired myelodysplastic syndromes, despite minor morphological changes (42). Contributions of Tp53 to terminal phagocyte differentiation and functional competence is of interest for future study.

Our *in vivo* studies associated lack of neutrophil accumulation during emergency granulopoiesis with impaired cell cycle checkpoint activity in *Fancc*^−/−^ or *Fancc*^−/−^*Tp53*^+/−^ mice, but not in WT mice. In WT or *Fancc*^−/−^*Tp53*^+/−^ mice with neutrophil accumulation, we found an increase in the percent of LSKs in G0/G1 during emergency granulopoiesis, and a decrease in the percent of LSKs in S or G2/M. This was consistent with G1/S arrest to resolve collapsed or stalled replication forks during the genotoxic stress of this process. Conversely, the percent of LSKs in G0/G1 decreased in *Fancc*^−/−^ mice during emergency granulopoiesis, with a relative increase in cells in S and G2/M. We hypothesize that this reflects an enhanced drive for neutrophil production during unsuccessful emergency granulopoiesis, despite DNA damage, in *Fancc*^−/−^ bone marrow. The intensity of EdU staining of individual *Fancc*^−/−^ LSKs cells was greater than *Fancc*^−/−^*Tp53*^+/−^ or WT cells, suggesting the increase in S phase in the former did not reflect activation of the intra-S checkpoint or overwhelming DNA-damage, but active DNA synthesis. Therefore, our data suggest a decrease in G1/S and G2/M checkpoints in *Fancc*^−/−^ mice during emergency granulopoiesis relative to WT or *Fancc*^−/−^*Tp53*^+/−^ mice. Cell cycle checkpoints were not different than WT in *Fancc*^−/−^*Tp53*^+/−^ mice during this process, suggesting use of a non-Tp53 mechanisms in the latter, such as Chk1.

During emergency granulopoiesis, we found increased Fbxw10 and decreased Fbxw11 expression in LSK transcriptomes from *Fancc*^−/−^ mice or *Fancc*^−/−^*Tp53*^+/−^ mice with blocked neutrophil accumulation, suggesting possible mechanisms for the observed cell cycle checkpoint disruptions. We also found decreased Atr protein and a relative decrease in phospho *versus* total Chk1 in *Fancc*^−/−^ bone marrow compared to WT (43). This might be consistent with increased Fbxw10 expression in *Fancc*^−/−^ bone marrow leading to ubiquitination and degradation of Atr and decreased Chk1 activity. To begin to connect increased Fbxw10 with loss of cell cycle checkpoints during emergency granulopoiesis, we knocked Fbxw10 down in in *Fancc*^−/−^ bone marrow 2 weeks after Alum injection. For these studies, we used a lentiviral vector to coexpress several Fbxw10-specific shRNAs and GFP. We found use of this vector to knockdown of Fbxw10 increased the percent of cells in G0/G1 with a relative decrease in S phase cells, consistent with possible rescue of the G1/S checkpoint. Further characterization is being pursued in the laboratory to clarify the mechanism for this Fbxw10-knockdown effect.

By LSK transcriptome analysis, we also associated impaired neutrophil accumulation during emergency granulopoiesis with decreased oxidative phosphorylation in *Fancc*^−/−^ *versus Fancc*^−/−^*Tp53*^+/−^ mice, *Fancc*^−/−^*Tp53*^+/−^ mice with Ly6G-treatment *versus* without Ly6G-treatment, or WT mice with *versus* without Ly6G, consistent with the metabolic stress of sustained inflammation. However, impaired neutrophil accumulation during emergency granulopoiesis induced an oxidative stress response only in WT LSKs. This raises the possibility that uncompensated bone marrow stress in FA during emergency granulopoiesis favors a switch to aerobic glycolysis, leading to HBP inhibition and a decrease in UDP-GlcNAc production (44). Perhaps, consistent with this, we also found an increase in lactate dehydrogenase A expression in LSK transcriptomes from *Fancc*^−/−^ mice during emergency granulopoiesis compared to LSKs from *Fancc*^−/−^*Tp53*^+/−^ or WT mice. Possible metabolic changes during the stress of emergency granulopoiesis potentially implied by these data are of interest for future studies.

Treatment with D-glucosamine or overexpression of Ogt inhibits *FBXW10* gene transcription, but not genes for other E3 ligases (44). We found increased Fbxw10 and decreased Ogt expression in LSKs from *Fancc*^−/−^ *versus Fancc*^−/−^*Tp53*^+/−^ mice, or *Fancc*^−/−^*Tp53*^+/−^ mice with Ly6G-treatment *versus* without Ly6G-treatment, but not in WT mice with *versus* without Ly6G antibody. And, we found Ogt mRNA abundance decreased during emergency granulopoiesis in *Fancc*^−/−^ or Ly6G antibody treated *Fancc*^−/−^*Tp53*^+/−^ LSKs, but increased modestly in cells from *Fancc*^−/−^*Tp53*^+/−^ or WT mice. We hypothesize that decreased UDP-GlcNAc plus impaired Ogt expression in *Fancc*^−/−^ mice might increase Fbxw10 expression and contribute to cell cycle checkpoint inhibition during emergency granulopoiesis. Investigating the functional contribution of these Ogt expression differences to transcription factor O-GlcNAcylation and *FBXW10* transcription is of interest.

In *Fancc*^−/−^ or WT mice, impaired neutrophil accumulation during emergency granulopoiesis induced a transcriptome of impaired HSC quiescence. This suggests that the potential for HSC exhaustion with sustained emergency granulopoiesis is not unique to FA. In general, fewer transcriptome differences were found with *versus* without Ly6G-treatment of WT mice during emergency granulopoiesis compared to *Fancc*^−/−^Tp53^+/−^ mice during this process, perhaps representing more efficient handling of stress in WT cells due to intact DNA repair.

These results have potential translational implications. Decreased Atr activity during emergency granulopoiesis in FA could be targeted with proteasome inhibitors (*i.e.*, bortezomib) for bone marrow protection (45). Proteasome inhibitors are nonspecific, but relatively nonmyelosuppressive. If metabolic changes during emergency granulopoiesis increase Fbxw10 expression in FA, use of D-glucosamine, which is nontoxic, inhibits Fbxw10 expression and has antiinflammatory effects, might be bone marrow protective (44).

## Experimental procedures

### Mice

*Fancc*^+/−^ mice were a gift from D.W. Clapp (Indiana University, Indianapolis, IN). Peripheral blood was obtained by tail vein phlebotomy for complete blood counts by automated cell counter and May-Grünewald-Giemsa-stained for hand counting of myeloid blasts (blinded for cohort, 300 cells/slide) (17, 18). Decalcified sternal bone marrow samples were stained with hematoxylin-eosin for WBC counts. Photomicrographs were captured by light microscopy (x40).

### Emergency granulopoiesis assay

Mice were injected IP every 4 weeks with Alum (aluminum chloride/ovalbumin) for emergency granulopoiesis, or saline as a steady state control. Alum was prepared and injected as described (16, 17, 18). Some cohorts were IP injected daily for 2 weeks with Ly6G antibody (clone 1A8, BioXcell) or isotype control (200 μg/day) followed by 3x per week for 2 weeks (29). Six mice were assigned per cohort with no blinding and no animals excluded from analysis.

### Flow cytometry

Bone marrow HSCs were identified by lineage negative (Lin^-^) selection (Lineage Depletion Kit, Miltenyi Biotec), followed by flow cytometry with anti-mouse FITC-Sca1 (eBioscience) or Brilliant Violet 421-CD117 (BD Biosciences). Apoptosis was determined by flow cytometry (Annexin V-PE Apoptosis Detection Kit, BD Biosciences) (18). Proliferation was determined by 5-ethynyl-2′-deoxyuridine labeling (Click-iT Assay System, Life Technologies) (19). Some cells were stained with phospho-H2AX PE antibody (Invitrogen) (19). Cell cycle was determined by flow cytometry for DNA content (FxCycle Violet Stain, Thermo Fisher Scientific).

### Murine bone marrow transduction

*Fancc*^−/−^ and WT total bone marrow mononuclear cells were obtained 2 weeks after injection of Alum or saline control. Cells were transduced with a retroviral vector with shRNAs specific to Fbxw10 or scrambled control shRNAs (pGFP-C-shLenti-FBXW10; OriGene) using a 16 h incubation protocol (46). GFP^+^LSK cells were analyzed by flow cytometry for cell cycle status within 24 h after harvest, as described above.

### Western blotting

Lin^-^ lysates (30 μg) were analyzed by SDS-PAGE and serially probed with antibodies to Atr, phospho-Chk1, Chk1 (Cell Signaling Technology) and α Tubulin (Proteintech). Three independent lysates were analyzed and representative blots shown.

### Quantitative real-time PCR

RNA was extracted with TRIzol reagent (Invitrogen). Primers were designed with Applied Biosystems software (https://www.thermofisher.com/us/en/home/life-science/sequencing/sanger-sequencing/pre-designed-primers-pcr-sanger-sequencing.html) and real-time PCR performed by the SYBR green “standard curve” method (16, 17, 18, 19). Three independent samples were evaluated in triplicate and normalized to Actin.

### Transcriptome analysis

Stranded total RNA-seq was conducted by Northwestern University NUSeq Core. Total RNA was checked for quality on an Agilent Bioanalyzer 2100 (Agilent BioTek) and quantified with Qubit fluorometer (Thermo Fisher Scientific). The Illumina TruSeq Stranded Total RNA Library Preparation Kit was used to prepare sequencing libraries (Illumina). Libraries were sequenced with an Illumina HiSeq 4000 Sequencer (single-end 50 bp reads). Read quality was evaluated using FastQC. Adapters were trimmed and poor quality or rRNA aligning reads filtered by Trim Galore (www.bioinformatics. babraham.ac.uk/projects/trim_galore/). Cleaned reads were aligned using STAR (32). Read counts were calculated using HTSeq-Counts in conjunction with a gene annotation file (useast.ensembl.org/index.html). Differential expression was determined with DESeq2 with cutoff for significant differences a false discovery rate-adjusted *p*-value < 0.05 (33). Metascape was used for pathway analysis (34).

### Statistical analysis

Significance was determined by unpaired, 2-tailed Student’s *t* test, ANOVA or log-rank analysis with SigmaPlot software (SPSS Inc, Chicago IL, https://grafiti.com/). *p* values <0.05 were considered significant. Error bars represent ± standard deviation.

### Study approval

Animal studies were approved by the IACUCs of Northwestern University and Jesse Brown VA Medical Center.

## Data availability

RNA-Seq data is available in the GEO repository (GEO Submission GSE267161) and NCBI tracking system (number 24638144). All other data are provided in the manuscript with original data available from e-eklund@northwestern.edu.

## Conflict of interest

The authors declare that they have no conflicts of interest with the contents of this article.
